# Managing Patients With Concurrent High Risk for Bleeding and Thromboembolic Events

**DOI:** 10.7759/cureus.53557

**Published:** 2024-02-04

**Authors:** Roland Fejes, Tamás Szűcsborus, András Czombos, Csaba Góg, Zoltán Ruzsa

**Affiliations:** 1 Department of Internal Medicine, Healthcare Centre of Hódmezővásárhely-Makó, Makó, HUN; 2 Division of Invasive Cardiology, University of Szeged, Szeged, HUN

**Keywords:** high-risk pulmonary embolism, acute coronary syndrome, massive upper gastrointestinal hemorrhage, myocardial infarction, antiplatelet therapy

## Abstract

The number of patients with high bleeding risk (HBR) and high thromboembolic risk (HTR) is increasing. Gastrointestinal bleeding (GIH), acute coronary syndrome (ACS), and pulmonary embolism (PE) are representative conditions due to HBR/HTR. Although these disorders are located at opposite ends of the same disease spectrum, this does not mean a patient with HBR cannot have a concomitant HTR. The clinical manifestation of these two risks mostly results in critically ill patients for whom management means a huge challenge. We have numerous well-structured guidelines about treating GIH, ACS, or PE, but the literature and recommendations about the concomitant onset of these diseases are limited. Expert recommendations suggest an integrative, comprehensive assessment of patient and intervention-related factors to decide on the antithrombotic regimen with the best clinical benefit by assessing thrombotic and bleeding risks. In general, if thrombotic factors predominate, a longer duration, more aggressive antithrombotic regimen should be planned, and if bleeding susceptibility is higher, a shorter duration, de-escalated regimen should be pursued. In this study, we aimed to explore the clinical dilemmas involved by presenting two cases with delicate management.

## Introduction

Upper gastrointestinal hemorrhage (UGIH) and major thromboembolic events, such as acute coronary syndrome (ACS) or pulmonary embolism (PE) are severe and highly disabling conditions [1,2]. While UGIH and ACS/PE are located at opposite ends of the same disease spectrum associated with the coagulation system, this does not mean that a patient with high bleeding risk (HBR) does not also have a concomitant high thromboembolic risk (HTR) [3]. This phenomenon can be seen even in such clinical scenarios when acute bleeding proves to be the controversial trigger for the thromboembolic event. Certainly, the coexistence of these diseases is quite rare, but most of these patients are in critical condition, require intensive care, and have an unacceptably high mortality rate - higher than expected when the two conditions occur separately [4].

While there is extensive literature on the management of UGIH that develops as a result of anticoagulation (AC) or dual antiplatelet therapy (DAPT), which remain fundamental treatments for thromboembolic events, there are limited data on ACS and PE that develop during or shortly after UGIH. This is true in the case of management of patients with concomitant HBR and HTR [5]. Therefore, in this study, we aimed to explore the clinical dilemmas related to the management of patients with coexisting HBR and HTR.

## Case presentation

Summary of case 1

**Figure 1:**
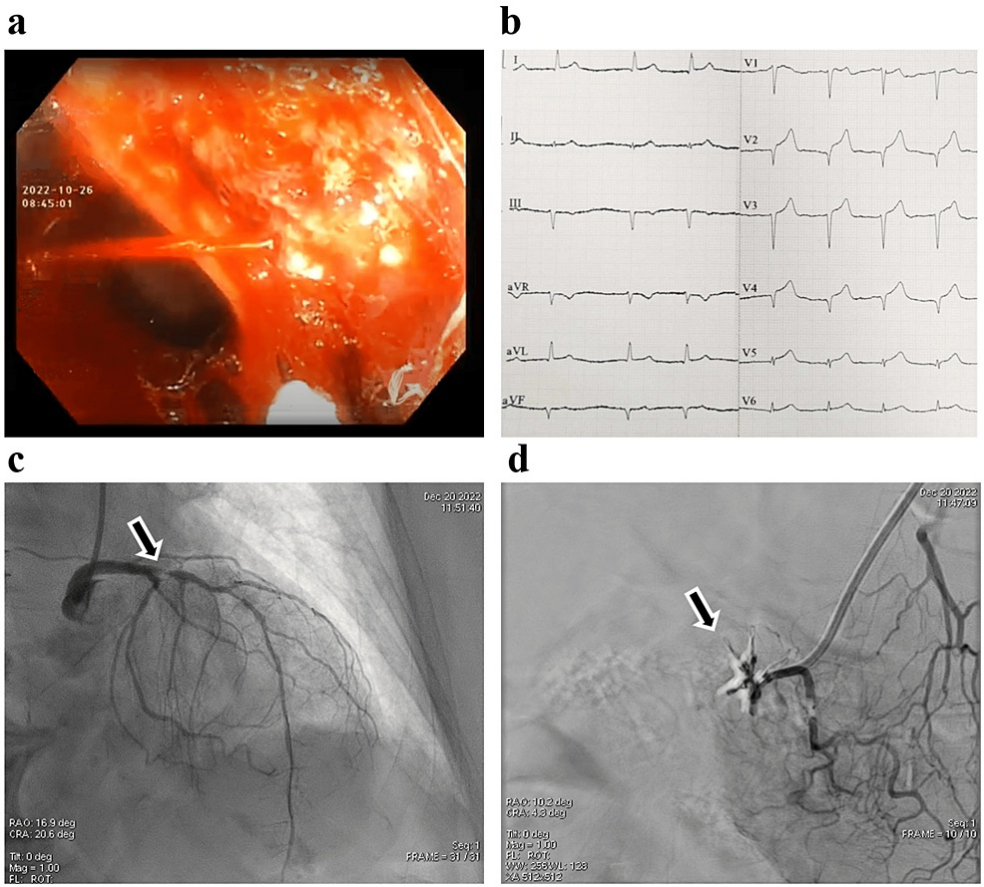
Clinical findings of the instability-causing lesions in case 1. (a) Forest I/a bleeding of the duodenal ulceration. (b) The ECG presenting ST-elevation acute coronary syndrome. (c) Left coronary artery angiogram. The black arrow points to the significant luminal stenosis responsible for cardiogenic shock. (d) Abdominal angiogram. The black arrow points to the puddle caused by the bleeding. Four pieces of hemoclips can be recognized that were applied to stop the hemorrhage.

An 80-year-old female patient was observed experiencing collapse, vomiting, and massive melena. Due to chronic joint pain, she had been taking metamizole tablets without any protection of gastric mucosa. She had no history of alcohol abuse or previous GIH and was not taking AC or antiplatelet therapy (APT) medication. Stable cardiopulmonary parameters were initially measured, and no relevant abnormalities were seen on the ECG. The patient presented a markedly painful epigastric region and an enormous amount of melena. Coffee ground-like gastric content was spontaneously evacuated without lavage through the nasogastric tube. The first 30 minutes of patient care involved triage, registration of initial vital signs and ECG, vein cannulation, blood sample collection, and transportation to a suitable observation room, during which a rapid hemorrhagic shock evolved (blood pressure: 75/40 mmHg; pulse: 120/min). The laboratory testing showed a normal level of cardiac necroenzymes and normocytic anemia with a Hb concentration of 7.8 g/dL (Table 1). Two months earlier the Hb level was 13.3 g/dL. Blood pressure targeted fluid resuscitation was started immediately and transfusion of two units of red blood cell concentration and intravenous administration of 80 mg pantoprazole, resulted in the stabilization of circulatory status. During pan-gastroscopy, a deep, 1-cm-wide duodenal ulcer with Forest I/a bleeding was discovered (Figure 1a) and treated through hemoclip application and adrenaline infiltration. Ten hours after the pan-endoscopy, the patient presented a new onset chest pain, with an anterior ST-elevation ACS (STE-ACS) on the ECG (Figure 1b) and markedly elevated cardiac necroenzyme levels (Table 1).

**Table 1:** Major laboratory findings and changes over time in case 1 and case 2. In both cases, laboratory values indicate the initial findings, when the patient was taken to our emergency department. In case 1, cardiac necroenzyme levels were controlled when new-onset chest pain was presented 10 hours after the endoscopic care. In case 2, only two hours passed between the Hb and Htc measurements. Reference ranges are indicated as used in the hospitals of the authors. Hb, hemoglobin; Htc, hematocrit; MCV, mean corpuscular volume; PLT, platelet count; INR, international normalized ratio; cTnI, cardiac troponin I; cTnT, cardiac troponin T; CK, creatinin-kinase; CK-MB, the fraction of creatinin-kinase MB isoform; paCO_2_, partial pressure of carbon dioxide in the arterial blood; pO_2_, partial pressure of oxygen in the arterial blood; SaO_2_, oxygen saturation of the arterial blood

	Laboratory findings in case 1	Laboratory findings in case 2	Reference range
Hb (g/dL)	7.8	7.7-6.0	12.0-15.0
Htc	0.22	0.21-0.18	0.35-0.45
MCV (fL)	101.5	87.6	80.0-95.0
PLT (G/L)	188	225	100-400
INR	1.1	1.0	0.8-1.1
D-dimer (μg/L)	672	6872	45.0-500.0
cTnI (ng/L)	7-6632	44	<19
cTnT (ng/L)	9-1114		<14
CK (U/L)	22-845	35	<180
CK-MB (%)	n/a to 12		<6
CRP (mg/L)	0.3	24	0.1-10.0
pH	7.35	7.47	7.35-7.45
paCO_2_ (mmHg)	30.8	32.7	35-40
paO_2_ (mmHg)	85.1	62.1	80-100
HCO_3_^-^ (mmol/L)	16.6	23.8	22-26
SaO_2_ (%)	96	93.5	>95

The case was presented to the invasive cardiologist in parallel with the transfusion of a further two units of red blood cell concentration. Although STE-ACS ceased during the transport to the invasive cardiology center, urgent coronary angiography was performed, during which a 98% stenosis was found in the proximal left anterior descending (LAD) coronary artery (Figure 1c). A delayed percutaneous coronary intervention (PCI) was planned, but after 12 hours cardiogenic shock evolved with no ST-segment elevation, so urgent PCI had to be carried out. To rule out active UGIH abdominal angiography was conducted, where no contrast leakage was observed in the gastroduodenal region (Figure 1d) and two drug-eluting stents were implanted in the LAD. Due to the intraprocedural cardiogenic shock with the lowest blood pressure value of 70/43 mmHg, intraoperative echocardiography was performed to rule out morphological complications. The left ventricular ejection fraction was estimated to be 48%. The distal 2/3 of the anterior wall and the apex appeared severely hypokinetic, and grade III mitral regurgitation was measured. Therefore, an intra-aortic balloon pump was inserted and successfully removed after two days. The initial APT included 100 mg aspirin once daily for three days, followed by DAPT comprising 100 mg aspirin once daily and 75 mg clopidogrel once daily. Additionally, gastric mucosal protection was provided with 40 mg pantoprazole twice daily. No bleeding was observed during subsequent patient monitoring, leading to discharge. DAPT was continued for one month, after which aspirin monotherapy was reinstated, and pantoprazole administration was maintained without discontinuation. The patient has been living an active life since then.

Summary of case 2

**Figure 2:**
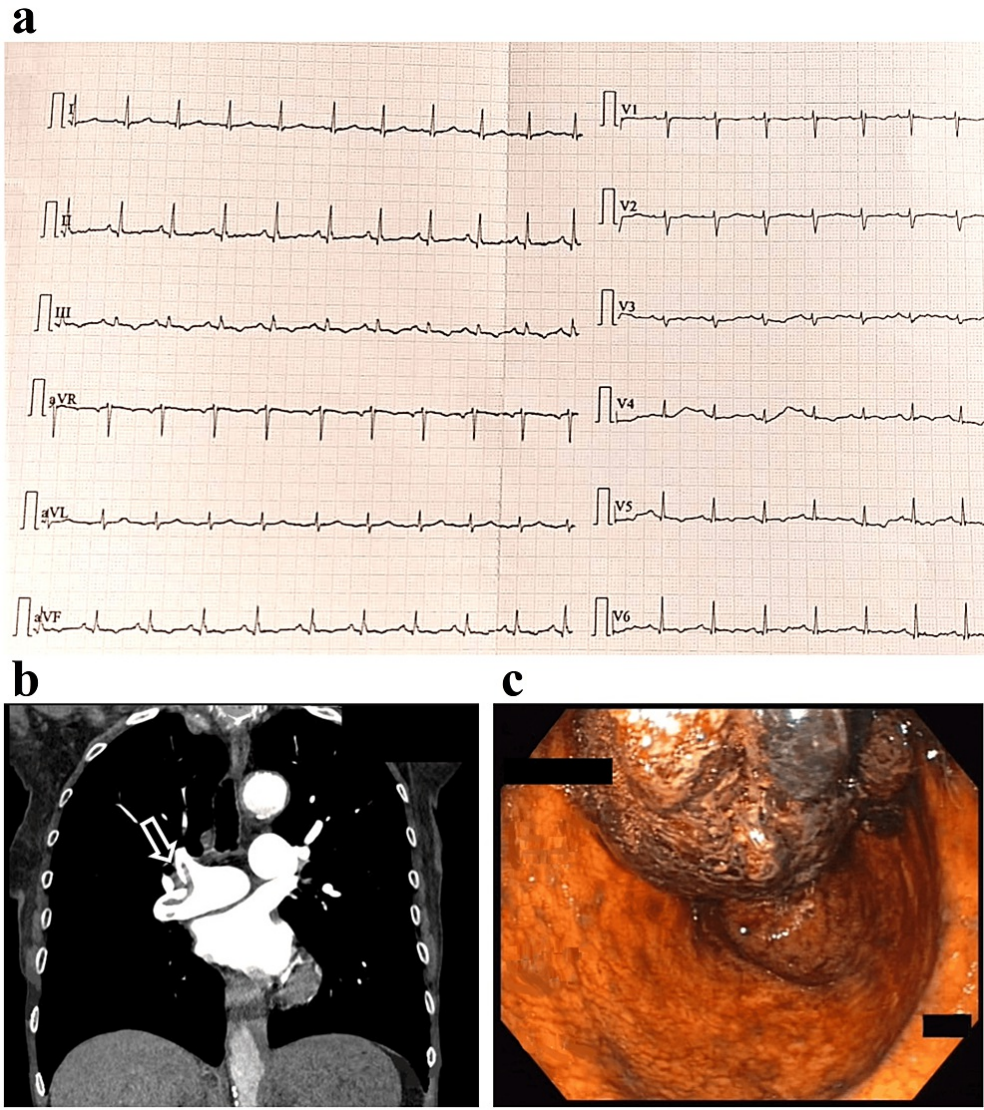
Major clinical findings of the instability-causing lesions in case 2. (a) ECG was taken due to the new onset dyspnea and chest pain. ECG presented sinus tachycardia with no other relevant abnormalities. (b) Pulmonary embolism in the branching of the right main pulmonary artery by CT pulmonary angiography. (c) A giant coagulum covering the gastric ulcer, which was the source of gastrointestinal bleeding. The bloody infiltration of the gastric mucosa is visible.

A 72-year-old female patient who underwent and completed complex radiochemotherapy for a nonmetastatic rectal tumor was observed in our emergency department with recent-onset chest pain, dyspnea, upper abdominal pain, and bloody stool from ileostomy. The physical examination did not show any notable findings, on her ECG a sinus tachycardia with a heart rate of 100-105 beats/minute was seen without any sign of myocardial ischemia (Figure 2a). Due to the elevated D-dimer levels observed in her laboratory results combined with a high thromboembolic risk (Wells score: 5 points) prompted a CT pulmonary angiography, which confirmed bilateral pulmonary embolism involving the right and left arches of the main pulmonary artery and three lobar branches on the right side (Figure 2b). Still in the emergency department, in the first one to two hours, rapidly progressive anemia was noted (Table 1), and due to the suspicion of UGIH, an upper gastrointestinal endoscopy was performed, revealing a large gastric ulcer with Forest I/a bleeding (Figure 2c). Successful hemostasis was achieved only on the second attempt. Thereafter the patient was admitted to the ICU with stable parameters, where, due to the high risk of bleeding, AC was achieved with a reduced dose of enoxaparin (4,000 IU q.d.). As a source of embolization extensive deep vein thrombosis was found in the left lower extremity. Considering the therapeutic protocols for concurrent thromboembolic and bleeding conditions, an inferior vena cava (IVC) filter was implanted after a risk-benefit assessment. Second-look endoscopy was carried out one week later and a more benign lesion was detected with no signs of active bleeding. Thereafter therapeutic AC was initiated by enoxaparin because a further operation was planned to remove the rectal tumor. An abdominal-pelvic MRI was performed, which confirmed the large-scale propagation of the tumor, because of which curative surgery was no longer possible. By the recommendation of the oncologist, a switch to oral anticoagulation was not conducted; therapeutic enoxaparin therapy was continued.

## Discussion

Considerations about case 1

**Figure 3:**
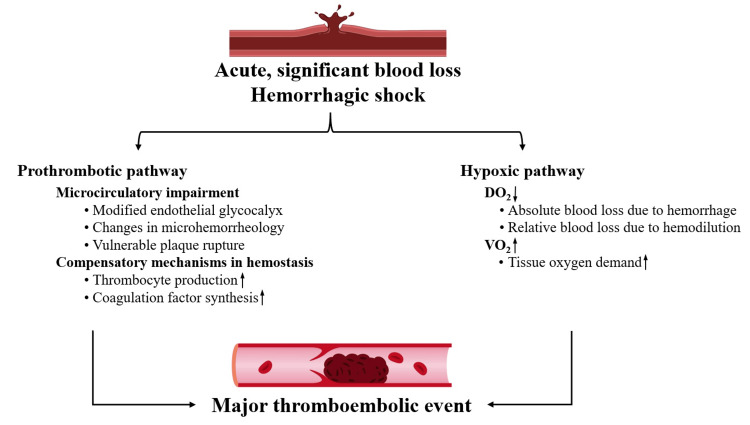
Major pathogenic routes of hemorrhagic shock leading to thromboembolic event. The pathogenesis involves not only the disruption of systemic and local regulation of hemostasis (left column) but also the consequential imbalance in oxygen dynamics (right column). Figure credit: Roland Fejes. DO_2_, oxygen supply; VO_2_, oxygen consumption

In case 1, the clinical course can be delineated into two distinct scenarios: hemorrhagic shock caused by UGIH followed by cardiogenic shock induced by ACS. The simultaneous occurrence of these two states is considered relatively rare; however, it must be anticipated due to the well-known physiological processes underlying it (Figure 3). Although these two manifestations presented in a time-dependent manner, it is imperative to acknowledge that HBR and HTR concurrently persisted throughout the entire management. In this perspective, the key to the adequate management of such patients may be found in perpetual risk assessment and prompt response. In connection with the use of a nasogastric (NG) tube, it must be mentioned that its routine use is not recommended by the European Society of Gastrointestinal Endoscopy [6]. Nonetheless, we believe that in certain urgent situations with a high time factor, the use of the NG tube can be a valuable tool for the differential diagnosis in the hands of healthcare personnel. Numerous scoring systems are known to detail the expected outcome, but a binary answer in terms of risk should always be made: HBR/HTR or not. Our patient presented with an overall Rockall score of 7 points, indicating a high risk of mortality and a heightened probability of requiring instrumental intervention [7]. Since blood loss can exacerbate ACS and may affect how PCI is performed in up to 40% of cases [8], we consider therapeutic endoscopy to be preferable over PCI if the source of instability is the bleeding, even if the diagnosis of ACS is established sooner. Otherwise, detecting ACS during UGIH involves many difficulties because a secure assessment of ischemic signs on ECG needs a stable hemodynamic status with a mean arterial pressure above 70 mmHg [9]. Indeed, if hemorrhagic and thrombotic risks are similar, hemorrhage is the dominant factor in terms of outcome and early treatment of bleeding should be considered. If the thrombotic and cardiac mortality risks are higher, PCI is preferred over other interventions [10].

Although STE-ACS was seen on the ECG, which requires immediate intervention according to the current recommendations at the time of the presentation of the case [11], the patient's complaints were due to anemia exacerbating severe coronary stenosis and resulting in non-ST-elevation ACS (NSTE-ACS). Since routine PCI for NSTE-ACS does not reduce overall mortality but increases the incidence of periprocedural complications, catheterization of patients at intermediate risk may be delayed up to 24 hours [12]. From a gastroenterological point of view, it is an important finding, as the first 72 hours after endoscopic intervention is the most critical period for ulcer healing [6]. It may be a further argument for early therapeutic endoscopy, because, as DAPT negatively affects GI mucosal healing, it seems to be a more appropriate procedure to perform endoscopy in DAPT naïve circumstances. PCI in high-risk NSTE-ACS should be performed within two hours, which was achieved in our case at the onset of cardiogenic shock - when the clot covering the ulcer had already had 40 hours to stabilize. Regarding the technique of PCI, total revascularization is superior to selective revascularization [13], yet in this case, dilating the vascular segment causing the acute problem avoided an unnecessarily high risk of bleeding and prolonged intervention. Additionally, it has to be also highlighted, that since routine use of IABP did not improve short and long-term outcome, it is not recommended by guidelines [6]. Yet the patient presented a rapidly developing cardiogenic shock and IABP was the fastest available opportunity for mechanical circulatory support with a lot of experience with its use, this might somewhat justify the legitimacy of the decision in this real-life emergency.

The main philosophic approach of postprocedural APT can be well described by the observation, that high bleeding risk negatively affects the expected outcome and therefore APT should be de-escalated as soon as possible, yet most thrombotic events occur shortly after PCI, making massive APT therapy essential [14]. It must be also added that the suspended clopidogrel and continuation of aspirin monotherapy are against the recommendations of the European Society of Cardiology. However, this question presents again the various, sometimes contradictory, professional considerations arising in connection with the case. Aspirin, which is a nonsteroidal anti-inflammatory drug, affects gastric mucosal healing to a greater extent compared to clopidogrel. Aspirin was found to be superior to clopidogrel in the prevention of recurrent gastrointestinal ulcer bleeding. We considered the patient to be at high risk for ulcer re-bleeding and the decision was made against the official cardiology guideline. Additionally, the European Society of Gastrointestinal Endoscopy does not recommend the suspension of aspirin used in secondary cardiac prevention in UGIH. The restart of DAPT is recommended immediately after hemorrhage in low-risk bleeding and according to relative risk ratios as soon as safe enough in case of high-risk bleeding. MASTER-DAPT trial means a cornerstone in long-term APT, thus in high-risk bleeding patients DAPT duration reduced to one month compared to conservative regimes was found to be non-inferior in terms of ischemic complications (6.1% vs. 5.9%) and superior (6.5% vs. 9.4%) in major or clinically relevant non-major bleeding [15].

Considerations about case 2

In case 2, we presented a patient in whom the underlying malignancy carried the risk of both HBR and HTR, and unfortunately, these risks manifested concurrently as two potentially life-threatening conditions. It is common, that the etiology of PE involves factors that make its treatment inadvisable. Furthermore, the likelihood of PE development is heightened in patients suffering from intercurrent illnesses. This means another circumstance that might also render conventional therapeutic approaches. The presented patient had a high-risk UGIH with a high-risk PE combined with HBR according to internationally accepted risk stratification scores (Rockall score: 7 points; VTE-BLEED: 3.5 points; Pulmonary Embolism Severity Index [PESI] score: 122 points) [16]. On this basis, the patient required endoscopic care, which although done successfully, still had too high a residual risk of bleeding due to its size and the diffuse nature of the hemorrhage. According to current guidelines, the treatment of low-risk PE patients with no signs of hemodynamic instability is AC by direct anticoagulants or by low-molecular-weight heparin [17]. In our presented case, the patient was cardiopulmonary stable with a high-risk and wide-extend embolism. In such circumstances, catheter-directed thrombolysis (CDT) or percutaneous pulmonary thrombectomy (PPT) are successful alternatives. CDT is recommended for patients with high-risk PE in whom thrombolysis is contraindicated due to HBR, or as rescue thrombolytic therapy for patients with hemodynamic deterioration on AC treatment. However, Hobohm et al. conducted a meta-analysis using data from 978.094 PE patients, and their results showed a significantly higher incidence of major bleeding complications alongside CDT, particularly among patients with malignancy [18]. Nevertheless, the supporting evidence for the recommendation for CDT is limited, and the technique continues to be a subject of debate due to the absence of sufficiently large trials [18]. Meanwhile, the multiplex localization of the embolization and the involvement of several vessels on both sides, suggest limited effectiveness about the technical part of PPT. It should be emphasized, that the large-scale and proximal deep vein thrombosis in the lower extremity meant an ongoing high risk of embolization. The solution to this would have been therapeutic AC but considering HBR, the risk-benefit assessment led to the exclusion of AC at therapeutic doses. Consequently, the decision was made to proceed with the insertion of an IVC filter to prevent further possible embolization. According to established guidelines, IVC filter placement is primarily indicated in cases where active bleeding with AC is impossible or poses an unacceptably high risk of bleeding. The current evidence with IVC filters is even more limited compared to CDT [19]. It is important to emphasize that IVC alone does not protect against the development of new-onset deep venous thrombosis, thus the initiation of AC should be expedited as soon as possible, especially in patients with malignancy, which means not transient but constant provoking factor for HTR [20]. This was the case in the presented scenario as well; a risk assessment performed during a subsequent endoscopy confirmed the resolution of HTR, allowing the initiation of therapeutic AC.

In our current understanding, the future of managing PE patients with HBR will likely rely increasingly on more refined catheter-based procedures. One of the main obstacles to this lies in the absence of large-scale studies within the broad population.

## Conclusions

Treating clinical conditions due to overlapping HBR and HTR erects numerous dilemmas, as we must act simultaneously at the two opposite points of the same disease spectrum. In an acute care setting, the priority is to stabilize the patient and fully assess the hemorrhagic and thrombotic risks to provide the most appropriate therapy. Long-term AC and APT regimens further complicate the management of such patients; therefore, more randomized controlled trials will be needed to ensure safer and more standardized care.
